# The chloroplast genome sequence of *Commelina communis* (Commelinaceae)

**DOI:** 10.1080/23802359.2019.1642153

**Published:** 2019-07-18

**Authors:** Yong Cui, Rifan Liang

**Affiliations:** a College of Horticulture and Landscape, Hunan Agricultural University, Changsha, China;; b Department of Agriculture and Environmental Engineering, Guangxi Vocational and Technical College, Nanning, China

**Keywords:** *Commelina communis*, complete chloroplast genome, automated assembly

## Abstract

*Commelina communis* is a hyperaccumulator on heavy metal with high drought resistance and endurance. Using Illumina next-generation sequencing data, its chloroplast genome is assembled and characterized. The complete chloroplast genome is 159,664 bp in length, consisting of a pair of inverted repeats (IRs) of 26,146 bp each, an 88,854 bp large single-copy (LSC) region and an 18,518 bp small single-copy (SSC) region. It has 137 genes in total, comprising 81 unique protein-coding genes, 32 unique tRNA genes, and 7 rRNA genes. Phylogenetic analysis based on the complete chloroplast genome sequences indicates that *C. communis* is sister to *Belosynapsis ciliata* among species with available chloroplast genome sequences.

*Commelina communis* L., an annual herb of Commelinaceae, can grow more vigorously on the heavy metal polluted field or mine (such as copper, cadmium, lead, uranium, etc.) than on the normal unpolluted soil, with larger biomass and higher growth rate (Wang et al. 2004; Zhang 2013; Lin et al. 2014; Liu et al. 2018). It can absorb the combined form of Cu^2+^ in the whole plant, while its roots can accumulate the highest concentration of Cu (Liao et al. 2003). This makes it an ideal candidate for ecological remediation of heavy metal contaminated soil or mining habitats. However, genomic resources of this species are extremely scarce. As a first step, here we aimed to assemble its complete chloroplast genome sequence.

An individual of *C. communis* was sampled in Tongan District, Xiamen, Fujian, China (118.15°E, 24.73°N), and a voucher specimen (Cui201903) was deposited at the Sun Yat-sen University Herbarium. Genomic DNA was extracted from fresh leaves using a modified CTAB method to prepare a DNA library prior to sequencing on an Illumina Hiseq X10 platform. We finally got 2 Gbp paired-end (length = 150 bp) sequence data, which were then fed into NOVOPlasty (Dierckxsens et al. 2017) to assemble the complete chloroplast genome sequence, using *rbc*L gene sequence of *C. communis* (GenBank accession No.: MK525584) as a seed with the seed-and-extend algorithm. Online software DOGMA (Wyman et al. 2004) was used to automatically annotate the chloroplast genome, coupled with manual double-check and adjustment. We then used OGDRAW (Lohse et al. 2007) to visual the gene map of the *C. communis* chloroplast genome.

The chloroplast genome of *C. communis* (GenBank accession No.: MK863371) possessed a typical quadripartite structure with a total length of 159,664 bp: two inverted repeats (IRs) of 26,146 bp each, an 88,854 bp large single-copy (LSC) region and an 18,518 bp small single-copy (SSC) region. It had 137 genes in total, comprising 81 unique protein-coding genes (among which seven are duplicated in the IR), 32 unique tRNA genes (seven are duplicated in the IR) and 7 rRNA genes. The genes *rps*19, *rps*16, *rpl*2, and *ndh*D had GTG, ATT, ATA, and ATC, respectively, as their start codons instead of the conventional ATG.

To infer the phylogenetic position of *C. communis*, a maximum-likelihood (ML) tree was constructed with 1000 bootstrap replicates using the GTRGAMMA substitution model (Stamatakis 2014), based on the complete chloroplast sequences of 16 species from four orders (Comelinales, Zingiberales, Poales, and Arecales) of commenlinids after aligning with MAFFT v7.307 (Katoh and Standley 2013). In addition, four other species from Aparagales were used as outgroups according to the APG IV (Angiosperm Phylogeny Group et al. 2016). As shown in Figure 1, *C. communis* is sister to *Belosynapsis ciliata* among these species. The complete chloroplast genome sequence of *C. communis* will provide useful genetic resources for future studies.

**Figure 1.**
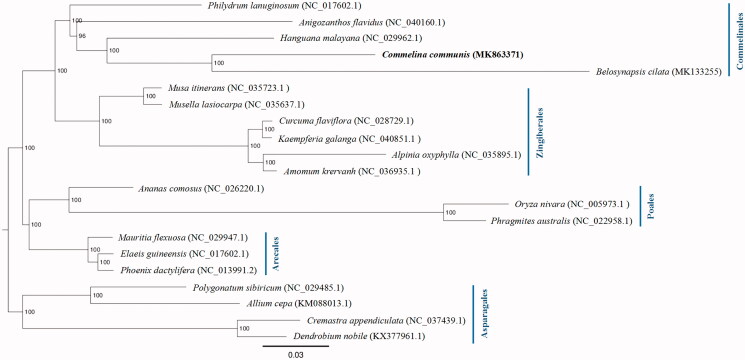
Maximum-likelihood tree showing the phylogenetic position of *Commelina communis* based on the complete chloroplast genome sequences. Bootstrap support values (based on 1000 replicates) are shown next to the nodes. Scale is substitutions per site.
